# Hydrogen Production through Catalytic Water Splitting Using Liquid-Phase Plasma over Bismuth Ferrite Catalyst

**DOI:** 10.3390/ijms222413591

**Published:** 2021-12-18

**Authors:** Kyong-Hwan Chung, Hyun-Hak Jung, Sun-Jae Kim, Young-Kwon Park, Sang-Chai Kim, Sang-Chul Jung

**Affiliations:** 1 Department of Environmental Engineering, Sunchon National University, Suncheon 57922, Korea; likeu21@hanmail.net (K.-H.C.); brand7911@nate.com (H.-H.J.); 2 Faculty of Nanotechnology and Advanced Materials Engineering, Sejong University, Seoul 05006, Korea; sjkim1@sejong.ac.kr; 3 School of Environmental Engineering, University of Seoul, Seoul 02504, Korea; catalica@uos.ac.kr; 4 Department of Environmental Education, Mokpo National University, Muan 58554, Korea; gikim@mokpo.ac.kr

**Keywords:** H_2_ production, water splitting, liquid-phase plasma, bismuth ferrite, optical emission

## Abstract

This study examined the H_2_ production characteristics from a decomposition reaction using liquid-phase plasma with a bismuth ferrite catalyst. The catalyst was prepared using a sol–gel reaction method. The physicochemical and optical properties of bismuth ferrite were analyzed. H_2_ production was carried out from a distilled water and aqueous methanol solution by direct irradiation via liquid-phase plasma. The catalyst absorbed visible-light over 610 nm. The measured bandgap of the bismuth ferrite was approximately 2.0 eV. The liquid-phase plasma emitted UV and visible-light simultaneously according to optical emission spectrometry. Bismuth ferrite induced a higher H_2_ production rate than the TiO_2_ photocatalyst because it responds to both UV and visible light generated from the liquid-phase plasma.

## 1. Introduction

Recently, as climate change has become more serious, measures have been sought to suppress the use of fossil fuels, which is the main cause of climate change, and to reduce the amount of carbon dioxide. Hydrogen energy is attracting attention as a clean alternative energy source that does not cause carbon dioxide or pollution. Therefore, there is a growing interest in expanding the use of hydrogen energy and securing more efficient hydrogen energy production technology. So far, most hydrogen production has been obtained from petroleum-based petrochemical processes or from steam reforming of methane or propane [1,2]. However, since these methods use hydrocarbons as starting materials, they release warming gases, such as carbon dioxide. Therefore, research on a hydrogen production process that does not emit carbon dioxide is receiving increasingly more attention [3,4]. Among them, hydrogen production by water electrolysis or photocatalytic decomposition is a clean technology that does not emit carbon dioxide. In addition, it can be recognized as a method of producing clean hydrogen when the electrical energy used in the reaction is obtained from renewable energy [5]. On the other hand, this method has a limitation in that the hydrogen productivity is low [6,7]. Therefore, an environmentally friendly way to produce hydrogen in large quantities has become the focus of research.

Since the discovery of photochemical reactions in which TiO_2_ electrodes decompose water to hydrogen and oxygen by photoelectrochemical reactions, various metal oxide photocatalysts have been developed [8,9,10,11,12]. TiO_2_ is chemically stable and has been studied extensively for photocatalytic reactions among them [13]. On the other hand, the quantum efficiency of TiO_2_ is limited by its wide bandgap and a high recombination rate of electron–hole. Therefore, a visible-light-responsive photocatalyst is essential for developing eco-technology by hydrogen production and organic matter cracking using solar energy. Nevertheless, the development of visible-light-sensitive photocatalysts is still at the research level and remains a major challenge.

A photocatalyst with high photo-reactivity can be produced by doping the TiO_2_ surface with metals or nonmetal ions, bonding to a semiconductor with a narrow bandgap, or doping with precious metals. Many studies have attempted to reform TiO_2_ [14,15,16]. The method of doping nitrogen on the TiO_2_ surface effectively reduces the bandgap by mixing the N 2*p* and O 2*p* states [17]. The photocatalyst efficiency was also shown to improve by doping the TiO_2_ structure with transition metals. Doping the TiO_2_ lattice with transition metals was shown to result in a red shift and enhance the photocatalytic activity [18]. The incorporation of the dopant increases or decreases the bandgap. The red-shift in the light absorption spectrum of Mn and N-doped TiO_2_ results in the formation of new energy levels between the Ti 3*d* state in the conduction band and the O 2*p* state in the valence band, resulting in a decrease in the bandgap [19].

Bismuth ferrite (BF) has ferromagnetic, ferroelectric, and hyperelastic properties [20], and has attracted considerable attention due to its high performance as a sensor material, data storage materials, and photovoltaic cells [21,22]. BF is a type of perovskite with a high ABO_3_ type and a multi-stiff material that exhibits both ferromagnetic and ferroelectric properties at room temperature [23]. Recently, it was reported that BF exhibits distinguish photo-catalytic activity in visible-light because of its narrow bandgap and high photovoltaic effects [24]. BF has a high response to visible light and is chemically stable. In addition, because BF nanocrystallines have magnetic semiconductor properties, they can be separated easily from solution [25]. In addition, the photovoltaic effects of BF also show increased reaction efficiency by inhibiting electron–hole recombination during the photochemical reaction [26]. Thus, BF is an interesting visible-light-sensitive photocatalyst that can be used for wastewater purification or H_2_ production from water using sunlight [27,28]. A method to enhance the photocatalytic activity of BF by doping the BF structure with rare earth elements has been studied [29].

This study examined hydrogen production from the decomposition reaction using a liquid-phase plasma (LPP) on a BF catalyst. The BF was prepared using a sol–gel method. The structural properties of the BFs prepared by varying calcining temperatures were investigated. The physicochemical and optical properties of BF were also analyzed. Hydrogen production was carried out in distilled water and aqueous methanol solutions by direct irradiation of LPP. The hydrogen production abilities were measured in various photocatalysts, including BF, and the results were studied in connection with the optical emission properties of LPP and absorption properties of light of the photocatalysts.

## 2. Results and Discussion

### 2.1. Physicochemical Properties of BF Photocatalysts

**Figure 1:**
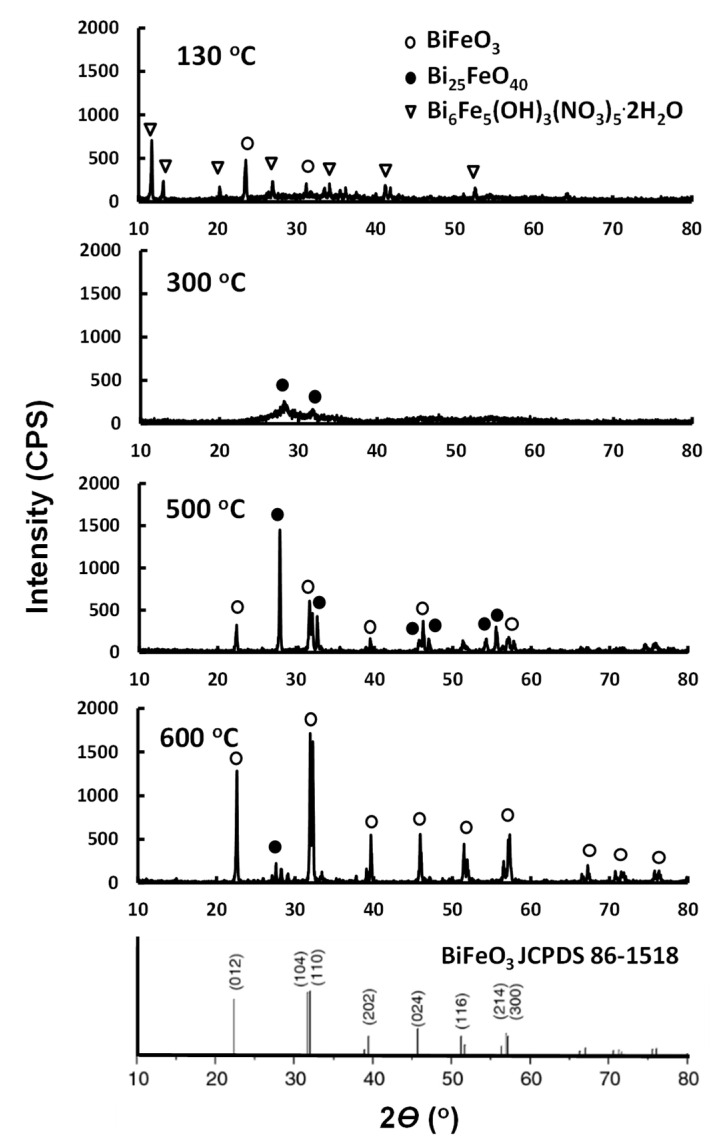
XRD patterns according to the heat treatment temperature.

Figure 1 presents the X-ray diffraction (XRD) patterns according to the heat treatment temperature of the BFs, which were heated at various temperatures. BiFeO_3_, Bi_24_Fe_2_O_39_, and Bi_6_O_5_(OH)_3_(NO_3_)_5_·2H_2_O structures were observed in the BF sample heat treated at 130 °C. BiFeO_3_, Bi_24_Fe_2_O_39_ and Bi_6_O_5_(OH)_3_(NO_3_)_5_·2H_2_O structures were compared with reference to JCPDS data [30,31]. The JCPDS data of BiFeO_3_, Bi_24_Fe_2_O_39,_ and Bi_6_O_5_(OH)_3_(NO_3_)_5_·2H_2_O structures are inserted in Figure 1. The standard XRD data of amorphous Bi_24_Fe_2_O_39_ was referred to in the literature [32]. When the BF was heat-treated at 300 °C, BiFeO_3_ and Bi_6_O_5_(OH)_3_(NO_3_)_5_·2H_2_O disappeared, and an amorphous Bi_24_Fe_2_O_39_ material was mainly observed. When the BF was calcined at 500 °C, a small amount of the Bi_24_Fe_2_O_39_ structure was observed, but it was mostly BiFeO_3_. When the BF was calcined at 600 °C, the peak representing the BiFeO_3_ structure became shaper and showed an almost pure BiFeO_3_ crystal structure.

Figure 1 shows the X-ray diffraction (XRD) patterns according to the annealing temperature of BF heated at various temperatures. In the BF sample annealed at 130 °C, the Bi_6_O_5_(OH)_3_(NO_3_)_5_∙2H_2_O structure was mainly observed. The structures of BiFeO_3_ (JCPDS 86-1518), Bi_25_FeO_40_ (JCPDS 46-0416), and Bi_6_O_5_(OH)_3_(NO_3_)_5_∙2H_2_O (ICSD 2406) were compared with reference to JCPDS data [30,31]. The JCPDS data of the BiFeO_3_ structure are inset in Figure 1. Standard XRD data of amorphous Bi_25_FeO_40_ were referenced in literature [32]. When BF was heat treated at 300 °C, Bi_6_O_5_(OH)_3_(NO_3_)_5_∙2H_2_O disappeared and amorphous Bi_25_FeO_40_ material was mainly observed. The intensity of the XRD peak was very low. When BF was calcined at 500 °C, a Bi_25_FeO_40_ peak and a BiFeO_3_ peak were mixed. When BF was calcined at 600 °C, the BiFeO_3_ structure was predominant, and the Bi_25_FeO_40_ peak was significantly reduced. It exhibits an almost pure BiFeO_3_ crystal structure.

**Figure 2:**
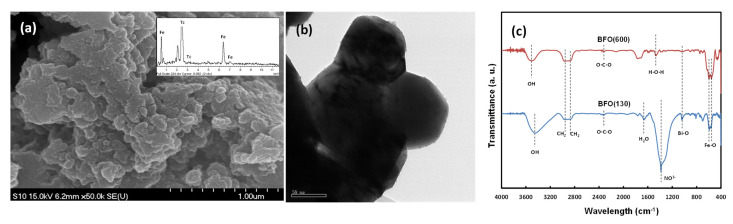
(**a**) SEM image with EDS results, (**b**) TEM image, and (**c**) FT-IR spectra of BF.

Figure 2a presents scanning electron microscopy (SEM) images and energy-dispersive X-ray spectroscopy (EDS) results of BF. Figure 2b presents transmission electron microscope (TEM) image of BF. The BF crystals showed irregular polygonal structures in the image. The size of the BF particles in the image was measured to be about 60 nm from TEM image. The particles appeared to be agglomerated with each other. The shape and size of the particles were similar regardless of the calcining temperature. The EDS revealed the two BFs to have similar components. The peaks for Fe were also observed in both EDS results. Figure 2c illustrates the FT-IR spectra of BF nanoparticles. The 594 cm^−1^ and 457 cm^−1^ peaks in the samples were assigned to the mode of stretching vibrations along the Fe-O axis and the mode of the Fe-O bending vibration, respectively [33]. This is the characteristic band exhibited by the FeO_6_ octahedron structure in the frame structure of the framework of perovskite [34]. The band at approximately 1070 cm^−1^ was ascribed to the Bi-O bonds vibration [35]. The broadband in the 3000–3500 cm^−1^ range was attributed to the symmetrical and asymmetric stretching vibrations of the H_2_O and OH bond groups, and the band of 1570–1600 cm^−1^ represents to the vibration of H_2_O bending [36]. The peak at 1380–1400 cm^−1^ was assigned to trace nitrates (NO^3−^) [37]. The band at 1574 cm^−1^ represents the H-O-H bending vibration. The peak at 2965 cm^−1^ is attributed to the asymmetric stretching of CH_2_ and the peak appearing at 3024 cm^−1^ is assigned to the symmetric stretching of CH_2_ [38].

**Figure 3:**
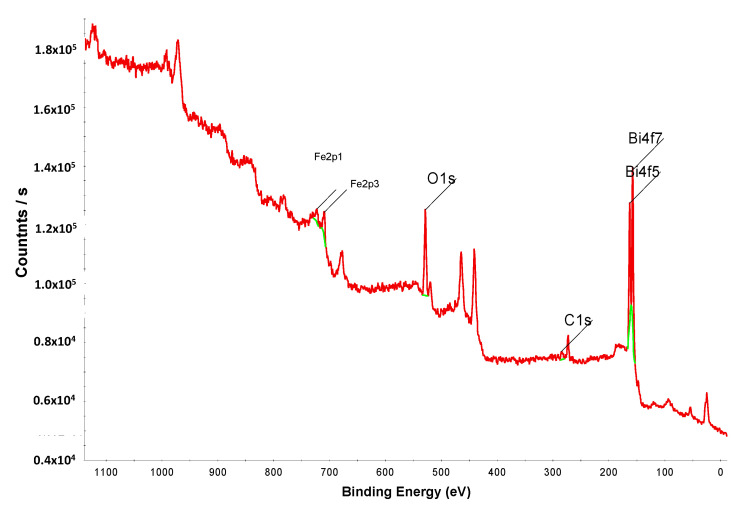
The entire XPS spectrum of BF.

X-ray photoelectron spectroscopy (XPS) was performed to confirm the surface state of the synthesized BF nanoparticles. Figure 3 illustrates the entire XPS spectra of the BF photocatalyst. The peaks for BF, such as Bi 4*f*_7_, Bi 4*f*_5_, Fe 2*p*_1_, C 1*s*, Fe 2*p*_3_, and O 1*s* were observed. The C 1*s* peak was also observed in the entire spectra, but the C adsorbed on the BF surface does not influence the physicochemical properties of the composites [39].

**Figure 4:**
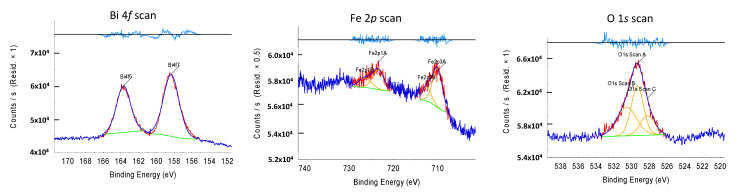
XPS spectra of Fe, Bi, and O for BF.

Figure 4 presents the XPS spectra of Bi, Fe, and O for BF. The individual spectrum of the Fe 2*p* core level was measured. The characteristic peaks of Fe 2*p*_1/2_ and Fe 2*p*_3/2_ appeared at 710.6 eV and 724.7 eV binding energies, respectively. This suggests that the Fe in the BF is in the Fe^3+^ state [40]. A weak satellite peak was obtained at 720 eV, indicating iron in the trivalent oxidation state and BF is BiFeO_3_ [41]. The peak near 530 eV in the O 1*s* spectrum was assigned to the oxygen interacting with Fe^3+^. The peaks of Bi were observed in the Bi element spectra. In the XPS results, the strong peaks of the spectra at 164 eV and 159 eV for Bi^3+^ were the Bi 4*f*_5/2_ and Bi 4*f*_7/2_ peaks [42]. The O 1*s* peak in the spectrum showed two kinds of chemical states of oxygen presents on BF. In the results, the peak at 529.3 eV binding energy was attributed to oxygen placed in the lattice, and the peak (~531.4 eV) corresponded to oxygen chemisorbed on the surface. The last peak was because BiFeO_3_-based compounds adsorb oxygen in the vacancies produced on the surface due to lattice defects [40].

**Figure 5:**
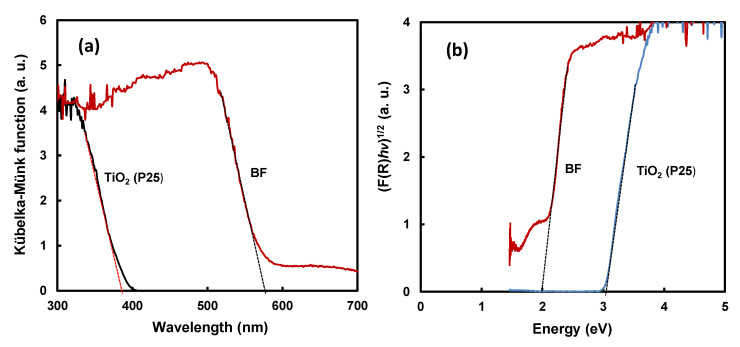
(**a**) Plot of the Kübelka–Münk function converted from UV-visible DRS and (**b**) relationship of (F(R)*hν*)^1/2^ versus energy.

Figure 5a shows a plot of the Kübelka–Münk function converted from UV-visible DRS of the BF [43]. BF absorbed ultraviolet and visible ray up to approximately 610 nm. The bandgaps of the BFs are determined from the plot of the Kübelka–Münk function (F(R)^1/2^), as shown in Figure 5b. The bandgap of BF photocatalyst was approximately 2.0 eV. The narrow bandgap of BF suggests that it can act photo-catalytic reaction, even in the visible-light region.

### 2.2. H_2_ production by the Photocatalytic Decomposition Using LPP

**Figure 6:**
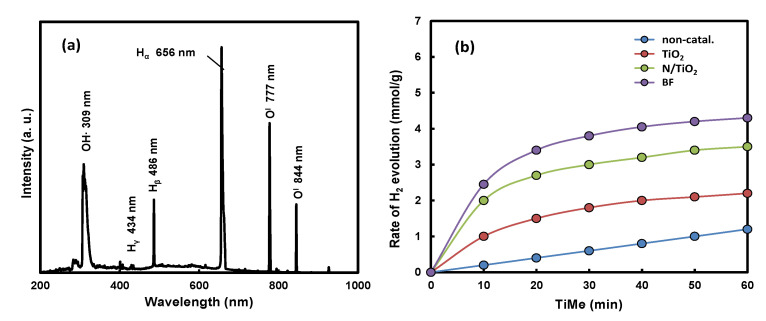
(**a**) OES spectrum of DW, and (**b**) HER under conditions without and with various catalysts.

Figure 6a shows the optical emission spectroscopy (OES) spectrum of DW. The OES of DW by LPP irradiation showed peaks at 309 nm, 486 nm, 656 nm, 777 nm, and 844 nm. Among the main peaks, the strongest peak appeared at 309 nm, which was an emission peak that appears along with the generation of OH radicals and shows strong oxidizing power [44]. In addition, it shows a strong emission peak at 656 nm. This emission peak appears along with H_2_ generation and is an emission peak that appears when active species decompose water to generate H_2_. The emission peak at 486 nm also appeared along with H_2_ production. The peaks at 777 nm and 844 nm were emission peaks appearing with oxygen production. On the other hand, the optical light emission by the LPP discharge in DW emits 309 nm-oriented UV light and visible-light of various wavelengths. Figure 6b shows the H_2_ evolution rate (HER) under conditions without and with various catalysts in H_2_ production from DW by LPP irradiation. H_2_ was generated by the cracking of water, even in the absence of a catalyst. As confirmed in the OES, the light at 486 nm and 656 nm was emitted by the LPP discharge to generate hydrogen. The HER was increased using a photocatalyst, such as TiO_2_. The HER was high in the order of BF > Ni/TiO_2_ > TiO_2_, which is related to the light absorption properties of the photocatalysts. According to the DRS results of each photocatalyst, TiO_2_ showed light absorption ability only up to UV light, but the light absorption band of Ni/TiO_2_ was expanded to the visible-ray region. The absorption region of BF was extended further into the visible-ray region. Therefore, in the Ni/TiO_2_ photocatalyst, a photoreaction occurred due to more light absorption than TiO_2_, increasing the HER. The HER was increased greatly because it can cause photochemical reactions by absorbing visible-light in a wider wavelength band than BF.

**Figure 7:**
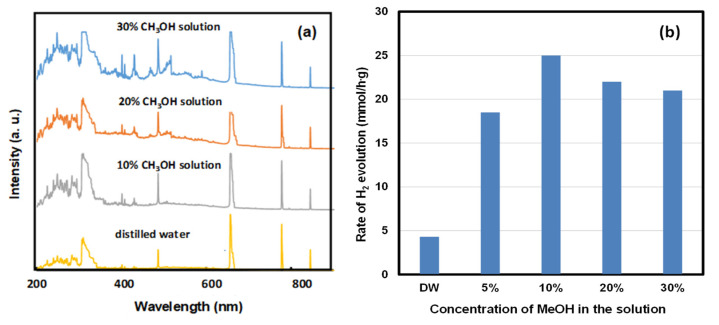
(**a**) OES spectra by LPP discharge and (**b**) HER of BF in an aqueous methanol solution with different concentrations.

Figure 7a illustrates the OES spectra by LPP discharge in an aqueous methanol solution with different concentrations. In the OES for an aqueous methanol solution, the optical emission peak at each wavelength was larger than the optical emission peak in DW. In particular, the optical emission peak of the wavelength in the UV region increased significantly. The OES spectra were studied in aqueous methanol solutions with different concentrations. The optical emission peak in the UV region increased gradually with increasing concentration but was highest when the methanol content was 10%. The emission peak decreased at higher concentrations. The size of the H^α^ (656 nm) peak was the same regardless of the methanol concentration. The size of the H^β^ peak at 486 nm slightly increased as the methanol concentration was increased. Figure 7b shows HER by the LPP irradiation and BF photocatalyst in an aqueous methanol solution with different concentrations. HER in the methanol solution was significantly higher than in DW. The highest HER was observed in a 10% aqueous methanol solution. The HER decreased gradually when the methanol concentration was higher than 10%. This is related to the change in OES according to the concentration of the aqueous methanol solution because UV emission is highest in an aqueous solution containing 10% methanol and decreases with increasing concentration. UV light emission was large in a 10% aqueous solution of methanol. Therefore, the photocatalytic decomposition of methanol and water occurred most actively, which explains why the HER was higher than in aqueous methanol at different concentrations.

## 3. Materials and Methods

### 3.1. Preparation of BF Nanoparticle Photocatalyst

The BF nanoparticles were prepared by a sol–gel reaction. The reagents were 0.05 M Bi(NO_3_)_3_·5H_2_O (Daejung, Gyeonggi-do, Korea, 99.5%), 0.05 M iron nitrate (Fe(NO_3_)_3_·9H_2_O; (Daejung, Gyeonggi-do, Korea, 99.5%), and 4.3 M C_2_H_5_OH (Daejung, Gyeonggi-do, Korea, 98%). The mixture of the reagents was stirred at 70 °C for 3 h to prepare a BF sol. The sol was dried at 150 °C for 1 day to prepare a powder, which was heated at 300 °C for 3 h and calcined at 600 °C for 4 h to get a BF powder. Commercial TiO_2_ (Evonik Degussa, Essen, Germany, P25) was applied as the control catalyst, Ni/TiO_2_ was prepared by doping Ni ions on TiO_2_ prepared using the sol–gel method [45].

### 3.2. Catalytic water Splitting Using LPP

**Figure 8:**
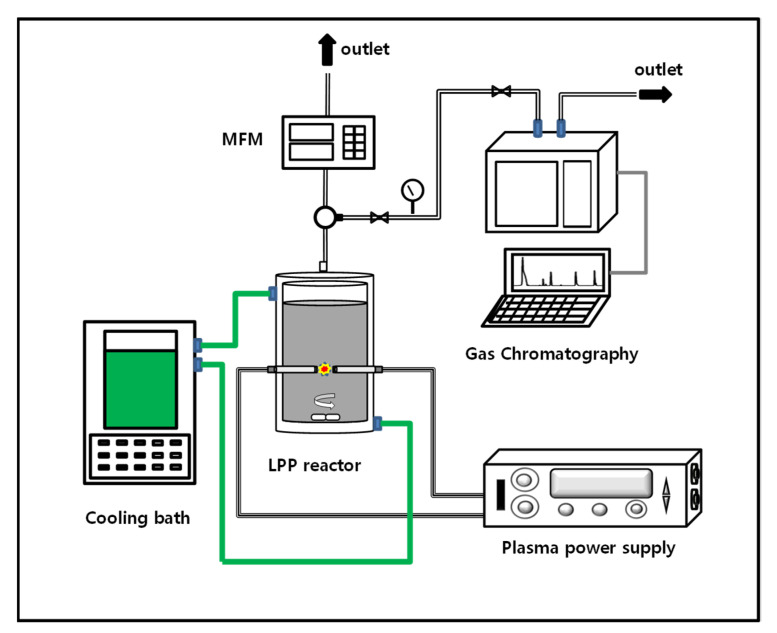
Schematic diagram of a photocatalytic reaction device using the LPP system.

Figure 8 shows a schematic diagram of a reactor for producing hydrogen by applying an LPP system. A catalyst (0.5 g) was injected into 100 mL of distilled water (DW) and reacted. The LPP reaction was carried out in a liquid mixed with the catalyst. The internal temperature of the reactor was maintained at 15 °C using a thermostatic circulator (Daehan, S2050, Seoul, Korea). The flow rate of hydrogen generated during the reaction was measured using a mass flow meter for measuring H_2_ gas (MFC Korea, TS-D2500, Buchon, Korea). The composition of the generated gas was analyzed by GC (Younglin, M700D, Anyang, Korea). Plasma was generated by the pulse discharge between the electrode and the electrode by the plasma generator [46]. Tungsten rods (D = 6 mm) were used as electrodes. The range of the plasma pulse width was 2–6 μs, and the frequency range of the plasma was adjusted to 20–35 kHz.

### 3.3. Characterization of BF Photocatalysts and Optical Emission of the LPP

The crystallinity and structure of the BF were observed by XRD system (Rigaku, D/Maxi Ultima III, Tokyo, Japan) using a CuKα X-ray radiation with Ni-filtered (λ = 1.5405 Å). The morphology and crystal size of the BF were surveyed by SEM system (Hitachi, S-4900/EXE-3000, Tokyo, Japan). The chemical compositions of the BF were surveyed by EDS system (NORANSE Z-MAX 550, Tokyo, Japan). FT-IR spectroscopy (Shimadzu, IRP resitge-21, Tokyo, Japan) of BF was performed to measure the functional groups. The binding state of the BF elements was investigated by XPS system (VG System, Multi-Lab 1000, East Grinstead, UK). Al Kα X-rays were used to measure the chamber at 10^−6^–10^−7^ Pa. The light absorption characteristics of the BF were analyzed by ultraviolet-visible DRS system (Shimadzu, UV-2750, Tokyo, Japan) using BaSO_4_ as a reflective standard to acquire the DRS band of the BF at 200–1000 nm. The optical bandgap of the BF was determined using the Kübelka–Münk theory [43].

The OES of the LPP was performed while the plasma was directly irradiating the reactants using a fiber optic spectrometer (Avante, AvanSpec-4500, Louisville, KY, USA). The OES spectra were gathered by placing an optical fiber perpendicular to the axis of the needle electrode [47]. The measurement conditions for gathering the spectrum were 3 μs pulse-width, 230 V discharge-voltage, and 35 kHz frequency.

## 4. Conclusions

The characteristics of H_2_ production by the catalyst of BF were studied in the cracking of water to which LPP was applied. BF absorbed visible-light over 610 nm. The bandgap of the BF photocatalyst was approximately 2.0 eV. LPP emitted ultraviolet and visible-light simultaneously. BF showed a higher H_2_ production rate than the TiO_2_ photocatalyst, which only absorbs ultraviolet light.

The HER was highest in the order of BF > Ni/TiO_2_ > TiO_2_. This is related to the light absorption properties of the photocatalyst. Although TiO_2_ showed light absorption only up to UV light, the light absorption band of Ni/TiO_2_ was expanded to the visible-ray region. The absorption range of BF was extended to the visible-ray region. The HER increased significantly because BF can cause a photochemical reaction by absorbing visible-light over a wider wavelength band.

In a methanol solution, the HER occurred at a significantly higher rate than in DW. The highest HER was observed in a 10% aqueous methanol solution. The HER decreased gradually when the methanol concentration was greater than 10% because UV emission was highest in the aqueous solution containing 10% methanol and decreased with increasing methanol concentration. Moreover, the photocatalytic decomposition of methanol and water occurred most actively in the 10% aqueous methanol solution because of the large UV light emission.

## Data Availability

Not applicable.
